# Editorial: Construction of nutrient delivery system for carotenoids and their antioxidant and anti-inflammatory activities

**DOI:** 10.3389/fnut.2024.1539429

**Published:** 2025-01-06

**Authors:** Jiangfeng Song, Yingdong Zhu, Jun Liu

**Affiliations:** ^1^Institute of Agro-Product Processing, Jiangsu Academy of Agricultural Sciences, Nanjing, China; ^2^Center for Excellence in Post-Harvest Technologies, North Carolina Agricultural and Technical State University, North Carolina Research Campus, Kannapolis, NC, United States; ^3^College of Food Science and Engineering, Yangzhou University, Yangzhou, China

**Keywords:** carotenoids, antioxidant, anti-inflammatory, gut microbiota, physiological pathways, potential mechanisms

## 1 Introduction

Carotenoids, a diverse group of natural pigments, have long been recognized for their potential role in promoting health and preventing disease. This Research Topic has brought together several studies that explore the various aspects of carotenoids, from their associations with mortality risk to their effects on specific health conditions. The studies presented here provide valuable insights into the complex relationship between carotenoids and human health, shedding light on both the potential benefits and the areas that require further investigation.

## 2 Carotenoids and mortality risk

The study by He et al. investigated the association between multiple serum carotenoid co-exposure and mortality risk in a large prospective cohort of US adults. Using data from the National Health and Nutrition Examination Survey (NHANES), the researchers found that higher serum levels of most carotenoids were associated with a reduced risk of all-cause, cardiovascular disease (CVD), and cancer mortality. The K-means clustering analysis identified different exposure patterns, with the high-level exposure group showing the lowest mortality risks. These findings suggest that a diet rich in diverse carotenoids may have significant health benefits, potentially through their antioxidant and anti-inflammatory properties.

## 3 Carotenoids and CVD mortality in metabolic syndrome

Han et al. focused on the impact of serum carotenoids on CVD mortality risk in middle-aged and elderly adults with metabolic syndrome. Their analysis of NHANES data revealed an inverse dose-response relationship between serum lycopene levels and CVD mortality risk. Interestingly, among the different carotenoids, only lycopene showed a significant inverse association with CVD mortality, while other carotenoids did not exhibit such a relationship. This study highlights the potential importance of lycopene in protecting against CVD in individuals with metabolic syndrome, a population at high risk for cardiovascular events.

## 4 Carotenoids and fecal incontinence

Li et al. explored the association between dietary carotenoid intake and fecal incontinence (FI) in American adults. Using data from NHANES, they found that higher intakes of carotenoids, particularly β-carotene and lutein/zeaxanthin, were associated with a reduced risk of FI. This is the first study to investigate this relationship, and the results suggest that dietary carotenoids may play a role in maintaining gut health and preventing FI. The mechanisms underlying this association may involve the anti-inflammatory and antioxidant properties of carotenoids, which could help to maintain the integrity of the intestinal barrier and modulate gut microbiota.

## 5 The broader role of dietary bioactive compounds and carotenoids in gut health

In addition to the specific studies on carotenoids, the review article by Wang et al. provides a comprehensive overview of the role of dietary bioactive compounds, including carotenoids, in modulating the gut microbiota and preventing and treating various diseases. The gut microbiota is emerging as a key player in human health, and dietary factors can significantly influence its composition and function. Bioactive compounds from vegetables, fruits, and other sources can regulate the gut microbiota, leading to the production of beneficial metabolites such as short-chain fatty acids, bile acid derivatives, and tryptophan metabolites. These metabolites, in turn, play crucial roles in maintaining gut homeostasis, influencing immune responses, energy metabolism, and inflammation. Understanding the complex interactions between dietary bioactive compounds and the gut microbiota is essential for developing strategies to prevent and treat diseases such as inflammatory bowel disease, colorectal cancer, CVD, obesity, and diabetes.

In the study by De Simoni et al., the authors conducted a narrative review to explore the role of antioxidant supplementation, including carotenoids, in the treatment of atopic dermatitis (AD). AD is a chronic inflammatory skin disease characterized by itching, epidermal barrier dysfunction, and an unbalanced inflammatory reaction. The review suggests that oxidative stress and lipid peroxidation contribute to the progression of inflammation in AD, and antioxidant agents may have a potential role in its prevention and treatment. Carotenoids, with their antioxidant and anti-inflammatory properties, could potentially be beneficial in managing AD. However, the clinical efficacy of dietary nutritional factors, including carotenoids, in the treatment of AD remains uncertain and requires further evaluation in clinical trials. The study also highlights the need for personalized approaches considering factors such as disease severity, comorbidities, and individual needs.

## 6 Future directions and implications

The studies presented in this Research Topic have several implications for future research and public health. Firstly, the consistent findings of an inverse association between carotenoid intake or serum levels and mortality risk, particularly CVD mortality, suggest that increasing carotenoid consumption may be a viable strategy for reducing the burden of chronic diseases. This could be achieved through dietary interventions, such as promoting the consumption of carotenoid-rich foods or developing functional foods and supplements.

Secondly, the differential effects of specific carotenoids, as seen in the studies on CVD mortality in metabolic syndrome and FI, highlight the need for a more detailed understanding of the mechanisms of action of individual carotenoids. Future research should focus on elucidating the specific pathways through which carotenoids exert their beneficial effects and identifying the factors that influence their bioavailability and efficacy.

Furthermore, the role of the gut microbiota in mediating the effects of carotenoids and other dietary bioactive compounds is an area of great interest. Understanding how these compounds interact with the gut microbiota and how these interactions impact health outcomes could lead to the development of personalized dietary interventions based on an individual's gut microbiota profile.

**Figure 1:**
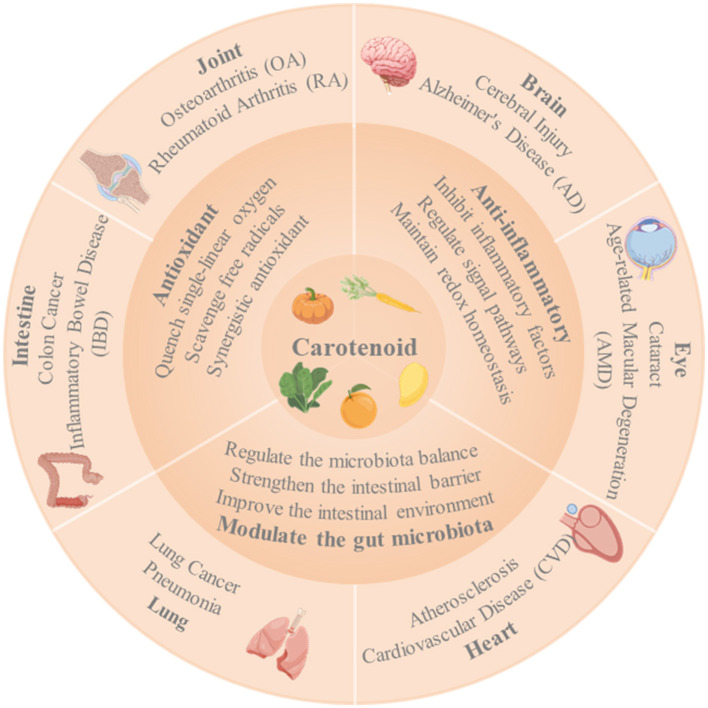
The potential mechanisms of action of carotenoids in health and disease, such as their antioxidant and anti-inflammatory effects, modulation of the gut microbiota, and impact on various physiological pathways.

In conclusion, the research presented in this Research Topic has significantly advanced our understanding of the role of carotenoids in health and disease. However, further studies are needed to fully elucidate the mechanisms underlying these associations (Figure 1) and to translate these findings into effective preventive and therapeutic strategies. By continuing to explore the potential of carotenoids and other dietary bioactive compounds, we can hope to unlock new avenues for promoting health and preventing disease in the future. This Research Topic serves as a valuable resource for researchers, clinicians, and health professionals interested in the role of nutrition in health and provides a foundation for future investigations in this exciting field. As we move forward, it will be important to consider the complex interactions between diet, the gut microbiota, and host metabolism to develop more targeted and effective interventions for improving public health. Additionally, further research is needed to determine the optimal doses and combinations of carotenoids and other bioactive compounds for maximum health benefits. With continued efforts, we may be able to harness the power of these natural compounds to improve the health and wellbeing of individuals around the world.

